# Asymmetry of VANGL2 in migrating lymphocytes as a tool to monitor activity of the mammalian WNT/planar cell polarity pathway

**DOI:** 10.1186/s12964-014-0079-1

**Published:** 2015-01-28

**Authors:** Markéta Kaucká, Julian Petersen, Pavlína Janovská, Tomasz Radaszkiewicz, Lucie Smyčková, Avais M Daulat, Jean-Paul Borg, Gunnar Schulte, Vitezslav Bryja

**Affiliations:** Institute of Experimental Biology, Faculty of Science, Masaryk University, Brno, Czech Republic; Department Physiology & Pharmacology, Sec Receptor Biology & Signaling, Karolinska Institutet, S-17177 Stockholm, Sweden; Centre de Recherche en Cancérologie de Marseille, INSERM U1068, F-13009 Marseille, France; Institut Paoli-Calmettes, F-13009 Marseille, France; Aix-Marseille Université, F-13284 Marseille, France; CNRS UMR7258, F-13009 Marseille, France; Department of Cytokinetics, Institute of Biophysics, Academy of Sciences of the Czech Republic, Brno, Czech Republic; Current address: Department Physiology & Pharmacology, Sec Developmental Biology and Regenerative Medicine, Karolinska Institutet, Stockholm, Sweden

**Keywords:** Planar cell polarity, Migration, B lymphocyte, VANGL2, Casein kinase 1, MEC1

## Abstract

**Background:**

The WNT/planar-cell-polarity (PCP) pathway is a key regulator of cell polarity and directional cell movements. Core PCP proteins such as Van Gogh-like2 (VANGL2) are evolutionarily highly conserved; however, the mammalian PCP machinery is still poorly understood mainly due to lack of suitable models and quantitative methodology. WNT/PCP has been implicated in many human diseases with the most distinguished positive role in the metastatic process, which accounts for more than 90% of cancer related deaths, and presents therefore an attractive target for pharmacological interventions. However, cellular assays for the assessment of PCP signaling, which would allow a more detailed mechanistic analysis of PCP function and possibly also high throughput screening for chemical compounds targeting mammalian PCP signaling, are still missing.

**Results:**

Here we describe a mammalian cell culture model, which correlates B lymphocyte migration of patient-derived MEC1 cells and asymmetric localization of fluorescently-tagged VANGL2. We show by live cell imaging that PCP proteins are polarized in MEC1 cells and that VANGL2 polarization is controlled by the same mechanism as in tissues i.e. it is dependent on casein kinase 1 activity. In addition, destruction of the actin cytoskeleton leads to migratory arrest and cell rounding while VANGL2-EGFP remains polarized suggesting that active PCP signaling visualized by polarized distribution of VANGL2 is a cause for and not a consequence of the asymmetric shape of a migrating cell.

**Conclusions:**

The presented imaging-based methodology allows overcoming limitations of earlier approaches to study the mammalian WNT/PCP pathway, which required *in vivo* models and analysis of complex tissues. Our system investigating PCP-like signaling on a single-cell level thus opens new possibilities for screening of compounds, which control asymmetric distribution of proteins in the PCP pathway.

**Electronic supplementary material:**

The online version of this article (doi:10.1186/s12964-014-0079-1) contains supplementary material, which is available to authorized users.

## Background

Cell polarization is a prerequisite for the control of cell shape, directional migration, asymmetric cell division, and cellular orientation in complex tissues. The WNT/planar-cell-polarity (PCP) pathway is crucial for the control of cell polarity. PCP pathway-mediated regulation of cell shape, directional migration, asymmetric cell division, and cellular orientation is required for normal development and function of complex tissues. Although evolutionary conserved core components of the PCP pathway and their functions were first identified in invertebrates and in lower vertebrates, the pathway plays comparably important roles also in human development [1,2]. Of particular interest, WNT/PCP has been implicated in many human diseases with the most distinguished positive role in the metastatic process, which accounts for more than 90% of cancer related deaths [3-6].

The function of the mammalian WNT/PCP machinery is still poorly understood and progress is hampered mostly by methodological barriers and limitations of non-mammalian experimental models. While the establishment of well-ordered ommatidia in the compound eye or cells in the wing epithelium in the fruitfly as well as convergent extension movements in *Xenopus laevis* gastrulation serve as important models of PCP signaling, assessment of PCP signaling in mammals is more difficult. In general it requires analysis of embryogenesis of mutant mouse strains where regular arrangement of sensory hair cells in the inner ear and neural tube closure phenotypes are the most commonly used readouts for PCP-like signaling in mammals [7,8]. However, cellular assays for the assessment of PCP signaling, which would allow a more detailed mechanistic analysis of PCP function and possibly also high throughput screening for chemical compounds targeting mammalian PCP signaling, are still missing.

Here we describe a novel mammalian cell culture model – the B lymphocyte-derived cell line MEC1 - suitable for analysis of PCP-like signaling on a single cell level. We employed live cell imaging and developed a novel and effective readout correlating subcellular localization of fluorescently-tagged PCP proteins, such as VANGL2, with MEC1 cell migration and chemotaxis. Importantly, asymmetric localization of VANGL2 in MEC1 cells is controlled by the same mechanisms as in mouse embryo as demonstrated by the requirement of casein kinase 1 (CK1)-mediated phosphorylation [9]. Our work advances the understanding of the PCP pathway beyond the borders defined by the powerful *Drosophila melanogaster* system, whose transferability is limited because of the evolutionary distance between the insect wing and compound eye to organs or cells found in mammals. Furthermore, this high throughput screen-compatible assay offers novel possibilities for quantitative assessment of mammalian PCP signaling and for the development of PCP-targeting drugs.

## Results and discussion

### MEC1 cells – a robust model for in vitro imaging of B cell chemotaxis

Our group has recently shown that the WNT/PCP pathway drives the pathogenesis of chronic lymphocytic leukemia (CLL) [10]. In that study we introduced the MEC1 cell model derived from transformed B cells of a CLL patient [11]. MEC1 cells recapitulate CLL behavior in many aspects and are used as a xenotransplantation model for CLL [12].

**Figure 1:**
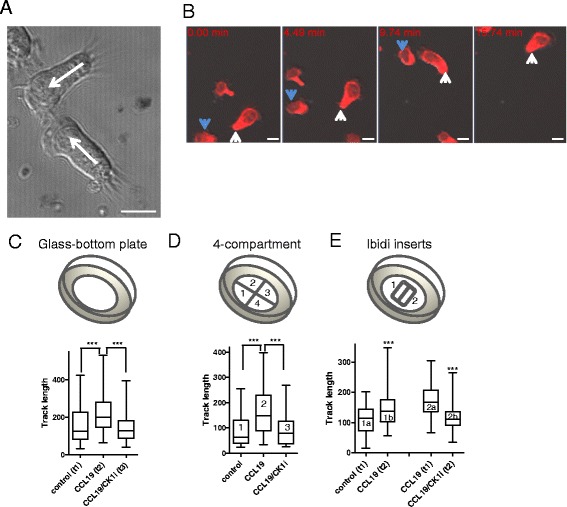
**Analysis of B lymphocyte (MEC1) cell migration by live cell imaging. (A)** A photomicrograph showing strongly polarized migrating MEC1 cells with the clearly defined leading and trailing edge. Arrows indicate the direction of migration. Size bar = 10 μm. **(B)** Snapshots of migrating, cell tracker-stained, MEC1 cells from time-lapse microscopy at approx. time: 0, 5, 10 and 15 min. Individual moving cells are indicated by the same color-coded arrow in each snapshot. Size bar = 10 μm. **(C)** MEC1 cells were seeded on glass-bottom plates coated with human plasma fibronectin. Control condition was captured first, for 30 min. Subsequently, CCL19 chemokine was added to the cells and the same position was scanned for additional 30 min. Last, cells were inhibited with PF670462 and followed another 30 min. **(D)** Four-compartment glass bottom plates were used for parallel tracking the cells (control [1], CCL19-treated [2] and CCL19/CK1 inhibitor-treated [3]). **(E)** Ibidi chamber for self-insertion was inserted in a glass-bottom plate, coated with Fibronectin and cell-tracker stained MEC1 cells were seeded. Ibidi chambers allow parallel scanning in two conditions (control [1a] and CCL19-stimulated [2a] first, captured for 30 min). After that, cells were stimulated with the chemokine [1b] and one of the pools was inhibited with the CK1 inh I [2b] and scanned for additional 30 min. From the first period, MEC1 migration in control and CCL19-stimulated wells [1a, 2a] is compared to each other and after that, CCL19 only and CCL19 + CK1 inh [1b, 2b] are compared to each other. At least 50 cells was tracked in each condition. ***, P < 0.001 (Kruskal-Wallis test).

MEC1 B lymphocytes cultivated on human plasma fibronectin-coated surfaces show the typical polarized morphology of a migrating cell with clearly defined leading and trailing edges (Figure 1A). MEC1 cells are capable to migrate efficiently as visualized by life cell imaging of MEC1 cells labeled with Cell Tracker™ Red CMTPX (time lapse image series in Figure 1B, Additional file 1: Movie 1). As seen in Figure 1B, MEC1 cells, approximately 15–20 μm in size, can move over the distance of their own size in less than 4 minutes. Importantly, due to their high motility, movies of migrating MEC1 cells are easily accessible to the automated computer-based quantification of migration parameters of individual cells.

Previously, we have described the basic parameters of MEC1 cell chemotaxis using transwell migration assays. In order to confirm that the dynamics in MEC1 migration seen in transwell assays is reproduced in the microscopy setting that we apply here, we seeded MEC1 cells in several other commercially available cell culture setups. The main characteristics of the MEC1 cells observed in the transwell model i.e. increased migration upon CCL19 and blockade of migration upon CK1 inhibition [10] were reproduced in all the tested setups. First, we have observed the effect in sequential treatments with control medium (30 min) followed with 30 min of CCL19 and subsequent CK1 inhibition (30 min) while imaging MEC1 migration in standard glass-bottom plates (Figure 1C). Second, we employed 4-compartment plates for simultaneous imaging of cells under different experimental conditions. The individual compartments contained control medium, CCL19 and CCL19/CK1 inhibitor (Figure 1D). Third, combination of the two approaches, i.e. simultaneous scanning followed by subsequent treatments is also possible as shown by the experiments in glass bottom cultivation plates with inserted two-chamber culture-insert from Ibidi (Munich, Germany) (Figure 1E).

**Figure 2:**
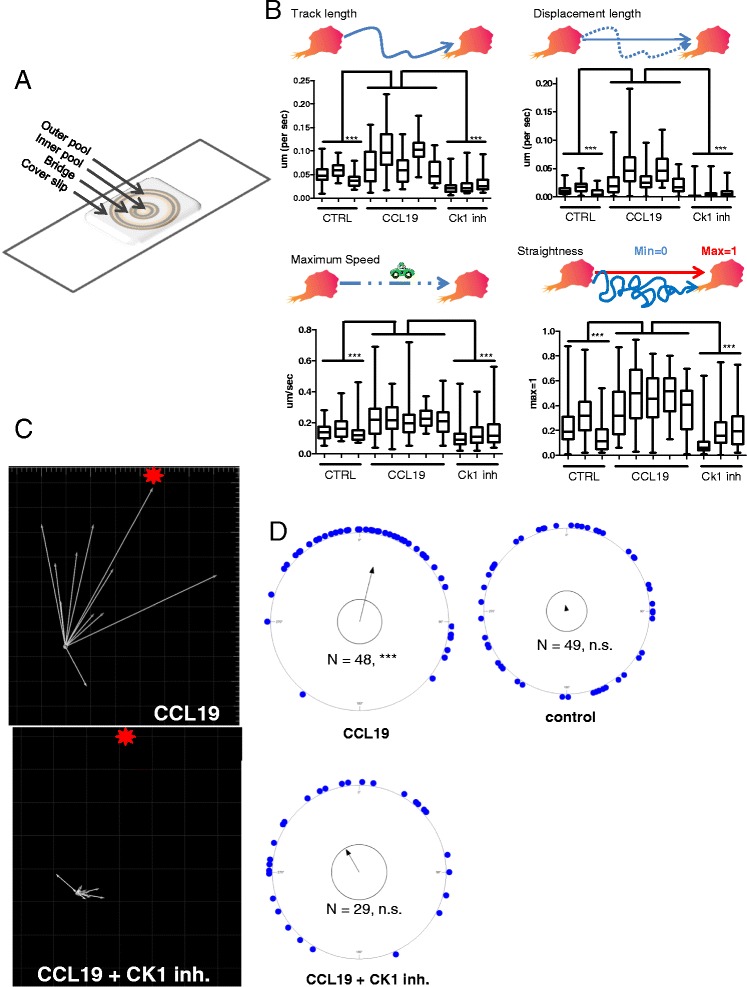
**MEC1 are suitable for analysis of directional chemotaxis.** **(A)** Dunn Chamber: the outer pool is filled with medium with/without chemokine, the inner pool is filled with medium only. Cells are scanned on the bridge between outer and inner pool. **(B)** Four graphs describe chemotaxis of MEC1 cells in a Dunn Chamber. Control (CTRL) - no chemokine gradient between two pools (n = 3). CCL19 gradient between inner and outer pool (n = 5). CK1 inhibition shows a condition with chemokine gradient between the pools in combination with CK1 inhibitor PF670642 (n = 3). Migration properties of tracked MEC1 cells are defined by Track length (exact distance of cell path), Track Displacement (distance of shortest path between each cell’s starting and the end point), Maximum Speed (maximum reached speed of each cell during the whole measurement) and Straightness (absolutely straight path = 1, minimum = 0). Statistical analysis was performed in GraphPad, using the Kruskal-Wallis test. (***, P < 0.001). **(C)** The representative example of trajectories of MEC1 cells in Dunn chamber-generated CCL19 gradient. Cells were treated with control solution (up) or CK1 inhibitor (bottom). The source of chemokine is indicated by a star. White arrows indicated the direction of migration by connecting the start and end point. **(D)** The statistical analysis of the directionality of migration was visualized using Oriana software. Individual blue dots (corresponding to individual cells) are plotted on the circle based on the deviation of their migration direction from the CCL19 source, which is set as 0° (up). Data were analyzed by the Rayleigh Test for randomness of circular data. Arrow points towards the predominant direction of cell migration; arrow length indicates statistical significance where only arrows crossing the inner circle (representing p = 0.05) are statistically significant. Please note that CCL19 adds a strong directional component to the migration, which is lost following CK1 inhibitor treatment. ***, P < 0.001.

Results in Figure 1 show that migration of MEC1 can be easily studied by live cell imaging. However, these experiments did not allow studying directionality of migration in the concentration gradient. In order to overcome this limitation we introduced Dunn chambers in the subsequent experiments [13], which were originally designed for imaging adherent cells migrating in a gradient (Figure 2A). The linear concentration gradient forms within Dunn chambers in approximately 20 min and is stable for up to 20 hours. The experiment shown in Figure 2B was performed repeatedly in three basic setups: (i) no chemokine (CTRL) in both pools (N = 3), (ii) chemokine CCL19 gradient with either 400 ng/ml CCL19 in the outer or inner pool to exclude loading artifacts (N = 5), and (iii) 400 ng/ml CCL19 in the outer pool plus casein kinase 1 inhibitor PF670462 (Tocris, 50 μM, N = 3). Cytotracker-stained cells were recorded by confocal live cell imaging in each condition and subsequently their migration properties (30–60 cells in each condition) were quantified using Bitplane Imaris Software 7.4 according to the procedures described in the M&M section (Figure 2B). Quantitative image analysis revealed that cells in the gradient of CCL19 migrated significantly longer distances compared to cells in control conditions (Figure 2B “Track length”). Furthermore, detailed analysis of the individual parameters of cell movement showed that the increased track length is a result of increased migration speed (Figure 2B “Speed max”) and straightness of the track (Figure 2B “Displacement Length” and “Straightness”). In addition, pharmacological inhibition of CK1 impeded CCL19-induced changes for all four migration parameters (Figure 2B).

Dunn chambers provide the unique possibility to analyze directionality of migration. Some of the migration parameters analyzed in Figure 2B (mainly “displacement length” or “straightness”) already allow to indirectly assess whether the directionality of migration is affected. In order to analyze the issue of directionality directly we plotted individual cell trajectories with respect to the source of chemokine (Figure 2C). Deviation of individual cells from the source of chemokine was plotted using Oriana software on the circular diagram and assessed statistically by Rayleigh’s test. As we show in Figure 2D, in the absence CCL19 cells migrate randomly whereas in presence of CCL19 gradient they coordinate and move towards the source of chemokine (P < 0.001). Inhibition of CK1 by D4476 affected not only the length of the trajectory but also its directionality, which is not significantly different from random migration (Figure 2D).

Thus, live cell imaging analysis of migration confirmed our observations seen in transwell assays. We demonstrated that migration of MEC1 cells can be analyzed by live imaging in several experimental setups, which allow both simultaneous analysis under several experimental conditions as well as analysis of consecutive treatments with several compounds in a time lapse manner. Importantly, the use of Dunn chambers allows performing delicate analysis of directionality of MEC1 migration. In summary, we provide protocols for novel, time-saving and robust methodology convenient for imaging of migration and chemotaxis of B lymphocytes.

### Migrating MEC1 cells show a polarized distribution of planar cell polarity proteins

**Figure 3:**
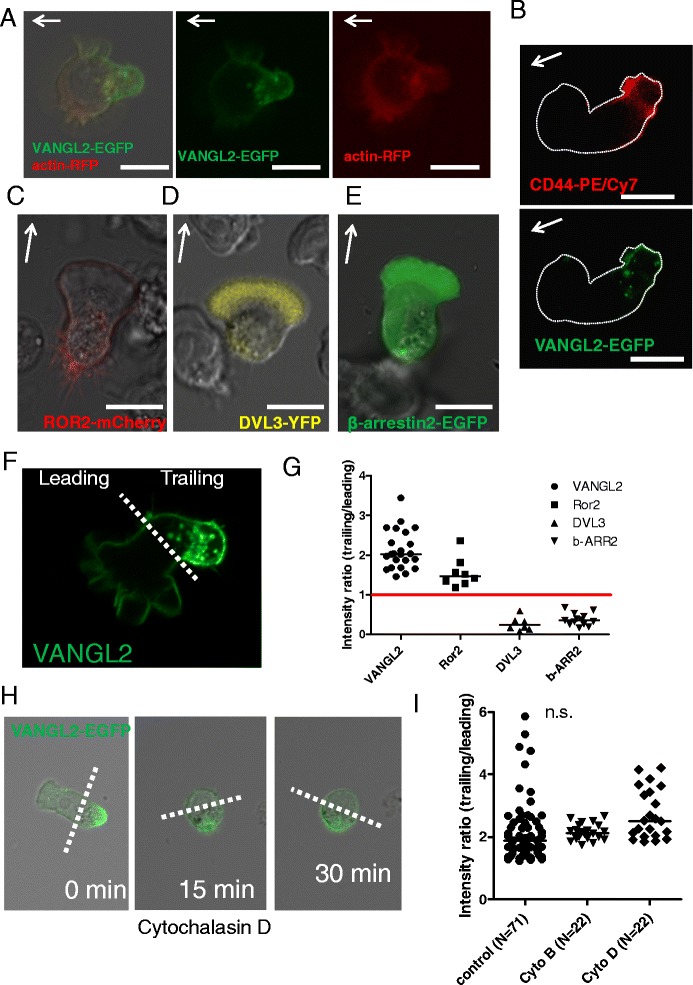
**Subcellular localization of fluorescently-tagged PCP proteins in polarized MEC1 cells. (A)** Photomicrograph of migrating polarized MEC1 cell with precisely defined leading and trailing edge. VANGL2-EGFP is significantly enriched in the trailing edge of the cell. To visualize the cytoskeleton, actin-RFP was co-transfected with the VANGL2 construct. Arrow indicates the direction of migration. Size bar = 10 μm. **(B)** Co-localization of EGFP-VANGL2 with endogenous CD44, a marker of the trailing edge. **(C,D,E)** Polarized structure of migrating MEC1 cells expressing ROR2-mCherry, DVL3-EYFP and β-arrestin 2-EGFP. Arrows indicate the direction of cell migration. Size bars = 10 μm. **(F)** To quantify the enrichment of VANGL2-EGFP in the trailing edge, the ratio of the EGFP signal intensity from leading and trailing edge was calculated using ImageJ software. Size bar = 10 μm. **(G)** Ratios of the signal intensity in the trailing edge divided by the signal intensity in the leading edge for all PCP proteins shown in A-E. One dot represents one analyzed cell. Red line indicates ideally symmetric distribution. **(H)** The effect of Cytochalasin D treatment on VANGL2-EGFP transfected MEC1 cell is shown after 0, 15 and 30 min. The localization of VANGL2-EGFP does not change even after the disruption of the actin cytoskeleton. **(I)** Quantification of individual cells recorded in E. The polarization of VANGL2-EGFP is expressed as the ratio between the EGFP signal in the trailing edge compared to the leading edge. Cytochalasin B and Cytochalasin D treatment does not affect the localization of VANGL2-EGFP in the MEC1 cell even though the typical migration morphology has been disrupted by the applied treatment. (n.s. – not significant).

We have shown earlier that migration of MEC1 cells (and CLL cells in general) depend on the PCP pathway and that cellular migration can be blocked e.g. by inhibition of CK1 or by downregulation of DVL2 [10]. Activity of the PCP pathway is reflected by the asymmetric subcellular distribution of the core PCP proteins [14]. In order to find out whether this feature is conserved in PCP-like signaling in B lymphocytes we took advantage of the novel model established in Figures 1 and 2. Cells were transiently transfected with various, fluorescently-tagged constructs encoding components of the PCP pathway, such as VANGL2, DVL3, ROR2, and β-arrestin. The most striking subcellular asymmetry in protein distribution was observed for the PCP protein VANGL2 (Figure 3A, co-transfected with actin-RFP to visualize cellular morphology). VANGL2-EGFP was distinctly enriched in the trailing edge, although low levels were also transiently observed in other cell regions. Most of VANGL2, a four transmembrane-spanning protein, was localized to the membrane and was enriched in the trailing edge (see 3D reconstruction of VANGL2-EGFP localization in Additional file 2: Movie 2) where it co-localized with the typical lymphocyte trailing edge marker CD44 [15] (Figure 3B). Enrichment in the trailing edge was observed also for the mCherry-tagged, single transmembrane-spanning WNT receptor ROR2 (Figure 3C). In contrast, two other, cytosolic proteins previously implicated in the PCP pathway – Dishevelled 3 (DVL3) and β-arrestin2 [16-18] – localized predominantly to the leading edge of the cell (Figure 3D and E). The level of asymmetry can be quantified by signal intensity measurements (schematized in Figure 3F) and the quantitative results for individual PCP proteins are presented in Figure 3G.

PCP proteins act as the determinants of cell polarity. As a next step we thus wanted to exclude the possibility that the polarized distribution of VANGL2 is simply a consequence of reorganization of actin cytoskeleton, which is dependent on the polarized cell morphology. We treated cells with the inhibitors of actin polymerization - cytochalasin D and cytochalasin B – and tested their effects on VANGL2 polarization. As we show in Figure 3H, and Additional file 3: Movie 3, destruction of the actin cytoskeleton by cytochalasin D (cyto D) treatment (15–30 min) leads to a dramatic change in cell shape – cells round up and stop moving. Interestingly, the VANGL2-EGFP stays asymmetrically distributed even after prolonged cyto D and cyto B treatment (3I). This observation demonstrates that key polarity determinants (such as VANGL2 in our case) are polarized even in completely round cells with compromised cytoskeletal functions, suggesting that polarized distribution of VANGL2 is a cause for and not a consequence of asymmetric shape of a migrating cell.

According to the best of our knowledge this is the first time that clear polarization of PCP proteins was observed *in vitro* in cells that are not embedded in tissue but rather lack cell-cell contact and grow unicellularly. It has to be mentioned, however, that PCP protein asymmetry has been recently reported in several cancer cell types with mesenchymal phenotype [3,19]. Our findings suggest that PCP proteins in MEC1 cells localize asymmetrically and recapitulate typical and conserved features of the core PCP protein localization from *Drosophila melanogaster*. Strikingly, this is despite the fact that B lymphocytes lack the persistent cell-cell interactions, which appear to be crucial for cellular asymmetry in two-dimensional tissues, such as the insect wing or eye. MEC1 cells can establish PCP-protein polarity despite the lack of extracellular Flamingo-Flamingo and Frizzled-VANGL interactions, which provide cell polarity information from one cell to its neighbor in insect tissues [1]. This raises the question whether the mechanism controlling VANGL2 asymmetry in MEC1 cells is similar to the mechanism controlling polarized localization of VANGL2 in tissues.

### Asymmetric localization of VANGL2 in migrating cells is controlled by casein kinase 1 (CK1)

**Figure 4:**
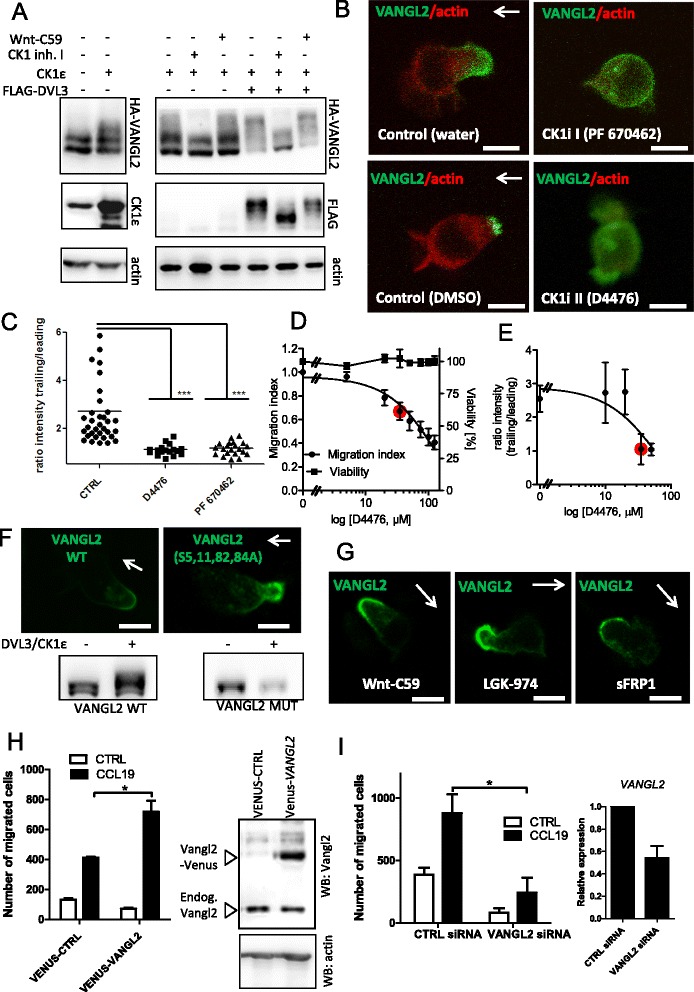
**Pharmacological inhibition of CK1 disturbs VANGL2-EGFP polarization. (A)** The effect of CK1ε, DVL3, CK1 inhibitor and Wnt-C59 (porcupine inhibitor) on the electrophoretic mobility of HA-VANGL2 as determined by Western blotting. **(B)** Snapshots from time-lapse microscopy of MEC1 cells co-transfected with VANGL2-EGFP and Actin-RFP. All conditions are CCL19-stimulated. Cells were treated with two casein kinase 1 inhibitors: PF670462 (CK1i-I) and D4476 (CK1i-II). **(C)** Ratios of the EGFP signal intensity in the trailing edge divided by the EGFP signal intensity in the leading edge for all three conditions are represented in the bar graph. Statistical analysis was performed using one-way ANOVA and Kruskal-Wallis test in GraphPad (***, P < 0.001). **(D,E)** Dose-dependent effects of D4476 (CK1i-II) on MEC1 cell migration, viability and VANGL2 asymmetry are summarized graphically. Migration (migration index) and asymmetry (ratio of fluorescent intensity in trailing/leading edge) were analysed by nonlinear regression. 35 μM D4476 is labelled red. Data points of viability experiments are shown with a connecting line (no regression). **(F,G)** Asymmetric distribution of VANLG2 in MEC1 cells is disturbed neither by the mutation of VANGL2 serines phosphorylated by CK1 to alanine (VANGL2-S5,8,11,82,84A) nor by inhibition of WNT production by porcupine inhibitors Wnt-C59 (10 μM) and LGK-974 (10 μM) nor by inhibition of WNTs by sFRP1 (10 μg/ml). The Western blot in F demonstrates that overexpression of CK1/DVL3 is unable to trigger phosphorylation-dependent shift of VANGL2-S5,8,11,82,84A. **(H)** Transwell migration assay experiment (N = 3) shows that VANGL2-Venus MEC1 cells respond significantly better to CCL19 stimuli. Number of migrated cells in 20 μL of sample is shown. Western blot confirms VANGL2-Venus in the stable cell line. **(I)** MEC1 cells nucleofected by VANGL2 siRNA show decrease in migration in conditions with and without CCL19. Expression of VANGL2 was significantly decreased 36 h after transfection as determined by qPCR. Migration in CCL19-treated conditions **(H,I)** was compared by unpaired t-test (*, P < 0.05).

In order to address this point we have decided to test, which molecular mechanisms control the asymmetric localization of PCP proteins in MEC1 cells. We have chosen the trailing edge-specific localization of VANGL2-EGFP, which shows the most striking unilateral enrichment, as the readout. It has been shown earlier that asymmetric localization of VANGL2 and its function in mammalian tissues depend on the phosphorylation gradient controlled by CK1 [9]. In line with that we show that CK1ε can promote, in a CK1-activity dependent manner, the phosphorylation dependent shift of HA-VANGL2 (Figure 4A). Importantly, inhibition of WNT secretion by the porcupine inhibitor Wnt-C59 [20] has no effect suggesting that CK1 can bypass eventual requirement for WNTs. On the other side, co-expression of DVL3, a crucial PCP component, dramatically promotes in synergy with CK1ε VANGL2 phosphorylation shift in a CK1-dependent manner (Figure 4A). In order to test whether a similar mechanism controls the polarized distribution of VANGL2 in MEC1 cells we have treated VANGL2-EGFP-expressing MEC1 cells with two chemically unrelated CK1-specific inhibitors PF670462 (CK1i I) and D4476 (CK1i II). As shown in Figure 4B, the treatment of CCL19-stimulated MEC1 cells with either of the CK1 inhibitors leads to the clear loss of the posterior accumulation of VANGL2. This effect is quantified by fluorescence intensity measurements in Figure 4C.

We hypothesize that asymmetric localization of PCP proteins correlates with directed migration of MEC1 cells. In order to address this question we treated MEC1 cells with the increasing doses of D4476 and analyzed the effects on migration index and asymmetric distribution of VANGL2. Even though nonlinear regression of the dose–response data did not allow exact definition of EC_50_ values due to a lack of data points reaching maximal inhibition at high D4476 concentrations we aimed at identifying an effective concentration of D4476 close to a halfmaximal effect. Based on the quantification of the inhibition of cell migration (Figure 4D) we obtained substantial but not maximal inhibition at 35 μM D4476 (marked in red). When quantifying VANGL2 polarization (Figure 4E) in response to increasing concentrations of D4476, 35 μM show a distinct effect which was not further accentuated by higher concentration (50 μM). This concentration range is in agreement with the original data describing the effects of D4476 on the CK1-mediated phosphorylation in cells [9,21,22]. Interestingly, mutation of VANGL2 N-terminal residues previously reported to be phosphorylated by CK1 – namely S5, S8, S11, S82 and S84 - to alanine (S5,8,11,82,84A) was not sufficient to disrupt its asymmetric localization in the trailing edge (Figure 4F). This suggests that other substrates of CK1 cooperate with VANGL2 and support its asymmetric distribution. DVL3 (see Figure 4A) and ROR2 [9], which both promote full VANGL2 phosphorylation and are well-defined CK1 targets and PCP proteins [23,24], are good candidates serving this function.

There is an ongoing debate in the field about the role of WNTs in the asymmetric localization of PCP proteins. In order to test if autocrine WNT signals could be required for maintenance of asymmetric localization of VANGL2 in our system we treated MEC1 cells with two porcupine inhibitors, LGK-974 [25] and Wnt-C59 [20] preventing WNT secretion, and with soluble Frizzled-related protein 1 (sFRP1) sequestering secreted WNTs. The inhibitors were used at concentrations that were previously described as effective [20,25,26]. Data presented in Figure 4G show that neither way of inhibiting autocrine WNT signaling disrupts the asymmetric localization of VANGL2.

As the last validation step of our model we wanted to analyze the role of VANGL2 in the migration of MEC1 cells. We generated a stable MEC1 cell line with Venus-VANGL2 and Venus-only expression as control. Comparison of migratory properties of these cell lines in transwell assays (Figure 4H) shows that Venus-VANGL2 MEC1 cells respond significantly better to the chemokine. In contrast, siRNA-mediated knockdown of *VANGL2* decreased the migratory properties of MEC1 cells (Figure 4I). These data demonstrate that VANGL2 is an important part of the chemotactic machinery that is both required for efficient migration and capable to promote chemotaxis when overexpressed.

We conclude that CK1-driven phosphorylation is required for posterior localization of VANGL2, which further correlates with the ability of MEC1 cells to directionally migrate towards the source of chemokine. As a consequence, we propose that parallel analysis of VANGL2-asymmetric localization (microscope) and chemotactic properties (transwell assay) can serve as a useful approach to discover novel regulators of PCP signaling.

Based on our findings we propose that microscopic analysis of the asymmetric distribution of VANGL2-EGFP is a robust and specific readout for the analysis of the PCP pathway. This analysis can be combined with the analysis of migratory properties of MEC1 cells although our results with the actin cytoskeleton blockers suggest that migration per se does not represent an ideal readout and can lead to false negative hits. Thus, we propose that the analysis of VANGL2-asymmetric localization using a microscope setup can serve as a useful approach to screen for novel small molecule compounds targeting mammalian PCP signaling. On the other hands the relative resistance of MEC1 cells to efficient transfection with siRNA (not shown) renders them less useful for siRNA screens, a limitation that might be overcome in foreseeable time by new developments in gene editing technologies.

PCP has been described and is still largely studied in the polarized epithelium in the insect wing or compound eye. This is mainly due to the combination of powerful fly genetics and relatively simple scoring of the phenotypes in fruitfly wings and eyes. The lack of asymmetry, determined either as the loss of asymmetric subcellular localization of PCP proteins or as the consequent defect in the positioning of wing bristles and/or individual ommatidia, has been shown to be a powerful tool for studying the PCP pathway in *Drosophila melanogaster* [27]. So far, a similar tool in mammalian cell culture systems has been unavailable. This is despite the assumption that the function of the core PCP module in vertebrates may differ from the mechanisms acting in the *Drosophila melanogaster* epithelium. Key PCP proteins are proposed to control mammalian cell shape changes (*e.g.* axon guidance), the positioning of basal bodies, primary cilium formation and asymmetric cell division (for recent review see [2]). This diversity and complexity implies that various accessory proteins “joined” the PCP pathway in mammals during evolution and that also core PCP protein modules might have been reorganized and modified for the sake of functional divergence and complex regulation in specialized cell types. Thus, experimental information gained in *Drosophila melanogaster* cannot necessarily be extrapolated to the mammalian concept of PCP-like communication.

The MEC1 model system described in this study, however, has the potential to bridge that gap and to contribute to the current toolboxes for the analysis of PCP (or PCP-related) pathways and processes in specialized mammalian cells. Our initial analysis suggests that the situation in MEC1 cells resembles in many aspects the core PCP features as established in *Drosophila melanogaster*. Similarly to most other experimental systems (for recent review see [2,28]) VANGL2 is localized on the opposite site of the cell than DVL, which suggests a high level of conservation of the basic PCP features. Our data also suggest that VANGL2 asymmetry cannot be disrupted by abolishing autocrine WNT signaling. However, we currently cannot exclude that WNTs are required for the establishment of the PCP protein asymmetry or that even low levels of WNTs present despite the use of porcupine inhibitors or sFRP1 are sufficient for maintenance of VANGL2 asymmetry. Our earlier work indeed showed that porcupine blockers can reduce MEC1 migration [10] but mechanistically it is not clear how. In vertebrate cells WNTs have been shown to be required for asymmetric localization of PCP proteins in many contexts including cells undergoing convergent extension [29,30], elongating muscle fibers [31] or melanoma cells [19]. Recent work implicated an instructive role of Wg and dWnt4 in PCP establishment in Drosophila and proposed a molecular mechanism how a WNT gradient [32] translates into the asymmetry of PCP proteins. The authors suggest that WNTs modulate the intercellular interaction of dFz and dVang in two neighboring cells, which is a mechanism that cannot apply to the single cell MEC1 system described in this study. We believe that further detailed analysis of the MEC1 experimental system will help to clarify the as yet still elusive role of WNTs in PCP [33] especially in the situation where cells are not embedded in an epithelial sheet.

## Conclusions

In summary, our work introduces a novel system for the analysis of asymmetric distribution of PCP proteins in relationship to PCP-dependent migration and chemotaxis. MEC1 cells may represent a desired model for the study of polarized distribution (and activity) of the WNT/PCP pathway in mammalian cell culture. Since MEC1 cells are migrating cancer cells, whose migration depends on WNT/PCP signaling [10] with strong polarization of VANGL2-EGFP, this model has the capacity to serve as a novel and long awaited high throughput screening platform. Adaptation of migration, chemotaxis and protein localization assays to multiwell plate format will allow for high throughput screening to identify compounds interfering with mammalian WNT/PCP signaling and for the study of key factors controlling the polarized distribution of PCP proteins and the activity of the PCP pathway as such. We believe that our novel model system has the potential to become the first assay, which allows unbiased analysis of PCP regulators/inhibitors in a cell culture assay. Compounds/genes identified in this essay can become a basis for novel therapeutics targeting PCP pathway in cancer and in metastatic process.

## Methods

### Culture and transfection of MEC1 cells

MEC1 cells were obtained from German Collection of Microorganisms and Cell Cultures (DSMZ). MEC1 were cultured in suspension in RPMI 1640 supplemented with 10% FBS and antibiotics at 37°C and 5% CO_2_. Transfection was performed using Amaxa™ 4D-Nucleofector™ system and P3 Primary Cell 4D-Nucleofector® X Kit and the EO-117 program according to manufacturer’s instructions. Each transfection reaction was performed with 1 × 10^6^ of MEC1 cells and 5 μg of DNA. After transfection 1 ml of pre-warmed medium was added into the nucleofection cuvette and cell were kept for 30 min at 37°C and 5% CO_2_ and only then transferred to the pre-warmed medium in T25 flasks. Transfected cells rested overnight before each experiment. For cell tracking, MEC1 cells were incubated for 1 hour at 37°C and 5% CO_2_ with CellTracker™ Red CMTPX at a final working concentration of 2.5 μM in serum-free RPMI medium. Subsequently, cells were centrifuged, washed with supplemented RPMI and used for live cell imaging.

Nucleofection of siRNA to MEC1 cells was performed by Neon transfection system (Life Technologies) according to the user’s manual. 10^6^ cells were transfected by 200nM CTR (Santa Cruz Biotechnology, sc-37007 ) or specific VANGL2 siRNA (Santa Cruz Biotechnology, sc-45595) in 10 μL reaction using buffer T. Cells were co-transfected by 0.5 ug of pmax-GFP per reaction to control transfection efficiency. After transfection, cells were incubated at least 24 h in full RPMI medium without antibiotics, then they were harvested for qPCR analysis of VANGL2 expression or seeded on transwell plate as described in following sections.

#### Lentivirus production and transductions

Lentiviral particules were produced using 293 T cells by polyethylenimine transfection of 3 μg VSV-G, 4 μg psPAX2 and 4 μg of either lenti-puro-VENUS or lenti-puro-VENUS-VANGL2 in 30-40% confluent monolayer cell culture grown in 10-cm Petri dishes. Media was changed 24 hours after transfection and new media was collected 24 hours later and filtered with syringe filter 0.22 μM. MEC1 cells were transduced in the presence of 10 μg/ml polybrene (Sigma-Aldrich). 24 hours after infection the viral media was replaced by fresh media. Transduced MEC1 cells were selected using puromycin (2 μg/mL) over a period of 8 days.

### Chemical inhibitors and plasmids

Casein kinase 1 inhibitor D4476 (4-[4-(2,3-Dihydro-1,4-benzodioxin-6-yl)-5-(2-pyridinyl)-1H-imidazol-2-yl]benzamide); casein kinase 1 inhibitor PF670462 (4-[1-Cyclohexyl-4-(4-fluorophenyl)-1H-imidazol-5-yl]-2-pyrimidinamine dihydrochloride); cytochalasin A (7(S)-hydroxy-16(R)-methyl-10-phenyl-24-oxa[14]cytochalasa-6(12),13(E),21(E)-triene-1,20,23-trione); cytochalasin D ((7S,13E,16S,18R,19E,21R)-21-(acetyloxy)-7,18-dihydroxy-16,18-dimethyl-10-phenyl[11]cytochalasa-6(12),13,19-triene-1,17-dione); porcupine inhibitor Wnt-C59 (4-(2-Methyl-4-pyridinyl)-*N*-[4-(3-pyridinyl)phenyl]benzeneacetamide), porcupine inhibitor LGK974 (2-(2',3-dimethyl-[2,4'-bipyridin]-5-yl)-N-(5-(pyrazin-2-yl)pyridin-2-yl)acetamide).

Plasmids used in the study that were described previously or commercially available: hVangl2-EGFP [34], xROR2-mCherry [35], β-arrestin-EGFP [36], mVangl2-HA [37], xCK1ε [38], hDvl3-flag [39], pDsRed-Monomer-Actin (Clontech), pmaxGFP (Lonza). hDvl3-EYFP was prepared by gateway cloning-mediated addition of EYFP on the C-terminus of Dvl3. Mutated Vangl2-EGFP was prepared by site directed mutagenesis of the hVangl2-EGFP vector (Quick change II XL site-directed mutagenesis kit, Agilent Technologies) of serins S5, 8, 11, 82 and 84 to alanins, using following mutagenesis primers: sense S5A 5'-ATGGACACCGAGGCCCAGTACTCGGG-3', antisense S5A: 5'-CCCGAGTACTGGGCCTCGGTGTCCAT-3'; sense S8A-S11A 5'-GAGGCCCAGTACGCGGGCTATGCCTACAAGTCGG-3´, antisense S8A-S11A 5'-CCGACTTGTAGGCATAGCCCGCGTACTGGGCCTC-3'; sense S82A-S84A 5'-GGCACCTCAGAGCAGGCCATCGCCCATGATGACCTCA-3', antisense S82A-S84A S82A-S84A: 5'-TGAGGTCATCATGGGCGATGGCGTGCTCTGAGGTGCC-3'. Vector used for MEC1 stable cell line preparation: cDNA of VANGL2 was amplified by PCR from the and flanked with Asc1/Not1 and cloned into lenti-puro-VENUS (Clonetech). All PCR-amplified region was verified by sequencing.

### Western blotting

For analysis of VANGL2 phosphorylation, HEK293 cells were seeded in density of 100 000 cells per well (24-well plate) and cultured in full DMEM medium (10% FBS, 1% P/S, 1% L-G; Biotech). Following day, they were transfected with total amount of 0.3 μg DNA per well using PEI transfection as described in [40]. After 6 h, medium was replaced by fresh one containing 20 μM CK1 inhibitor (PF-670462, Tocris), 10 μM Wnt-C59 (ab-142216, Abcam) or corresponding amount of MQ water and then the cells were incubated for another 24 h. Then they were lysed by 150 μL of 1% Laemmli buffer per well, sonicated and heated to 98°C for 2 min. VANGL2-Venus MEC1 cells were peleted (10^6^ cells per sample, 200 × g, 5 min, RT), washed by PBS and directly lysed by 1% Laemmli lysis byffer. Western blot analysis was done according to standard protocol, loading 25 μg of protein per sample. Proteins were separated by SDS-PAGE and transferred onto a PDVF membrane (Biotech), probed with the following primary antibodies: anti-Vangl2 (2G4, [41]), anti-actin (C-11, Santa Cruz Biotechnology, sc-1615), anti-flag (Sigma-Aldrich, FlagM2 F1804), anti-HA (Covance, MMS-101R), anti-CK1epsilon (C-20, Santa Cruz Biotechnology, sc-6471). The secondary antibodies were used as follows: Anti-Goat IgG (whole molecule) – Peroxidase antibody produced in rabbit (Sigma-Aldrich, A4174), Anti-Mouse IgG – Peroxidase antibody produced in sheep (Sigma-Aldrich, A6782) and Anti-Rat IgG − Peroxidase antibody produced in goat (Sigma-Aldrich, A9037). Signal was detected by the Fusion system for western blotting (Vilber Lourmat), Immobilon Western Chemiluminescent HRP Substrate (Millipore) was used.

### Direct immunocytochemistry

MEC1 cells were nucleofected by Neon transfection system (Life Technologies) as described above to express VANGL2-EGFP construct. After the transfection, cells were incubated for 24 h in full RMPI medium without antibiotics, then they were seeded on fibronectin coated glass coverslips (10 μg/mL fibronectin for 1 h, then blocked by full RPMI medium for 30 min before seeding cells) in a 24-well plate using complete RPMI medium. Cells were left to attach to the surface for 2 h. Then the unattached cells were removed together with medium. Coverslip with cells was washed carefully with PBS and cells were fixed by 4% PFA solution in PBS (15 min, RT). PFA was removed, cells washed twice with PBS and then PBTA solution containing conjugated primary CD44-PE/Cy7 antibody (Life Technologies, A16241) was added. Cells were incubated ON at +4°C in the dark, following day they were washed twice with PBS and the coverslip was mounted to glass slide by glycerol-gelatine (Sigma Aldrich, GG1-15 mL). Co-localisation of CD44-PE/Cy7 and VANGL2-EGFP signals in plasma membrane was then analyzed by confocal microscopy (Olympus FluoView 500).

### qPCR

Efficiency of VANGL2 siRNA knock-down in MEC1 cels was assessed by qPCR analysis of nucleofected samples at least 36 h after transfection. 10^6^ cells were peleted by centrifugation (200 × g, RT) and RNA was isolated by RNeasy extraction kit (Quigen). Reverse transcription was performed using oligo (dT) primer, dNTP mix and M-MuLV reverse transcriptase (all Thermo Scientific). *VANGL2* and actin (housekeeping gene as a control) expression was measured using LightCycler® 480 SYBR Green I Master and primers described previously [10].

### Confocal time lapse microscopy

Images and time series were acquired using a Zeiss LSM710 inverted laser-scanning microscope with Plan-Apochromat 20× objective. During time-lapse microscopy, several positions were captured every 20–40 seconds, depending on the type of experiment and number of followed positions.

For quantification of fluorescence intensity in the MEC1 cells, the ImageJ software (NIH, Washington, USA) was used. Based on our knowledge about the migration structure of MEC1 cells, we divided each cell into front (leading) and rear (trailing) part and we measured the intensity of the green channel in two regions-of-interest (ROI) of the same size. Subsequently, we calculated the ratio R of the signal intensity I as R = I_trailing_/I_leading_.

### Analysis of MEC1 migration

MEC1 cells were stained with CellTracker™ Red CMTPX (Invitrogen) according to manufacturer’s instructions for 1 hour as described above and washed once using cultivation medium. For the analysis of migratory properties of MEC1 cells in the CCL19 gradient, we used the Dunn chemotaxis chamber (Hawksley, UK). First of all, the glass coverslip, provided together with the chamber, was coated with human plasma fibronectin (10 μg/mL) for 1 hour at RT. Subsequently the coverslip was washed and incubated for another 30 min with FBS-supplemented RPMI medium. The CellTracker™ Red CMTPX-stained MEC1 cells were seeded on the coverslip and incubated for 45–60 minutes at 37°C and 5% CO_2_. Afterwards, unattached cells were gently washed away with the cultivation medium and exceeding medium was removed by slightly inclining the cover-slip and using the paper tissue to soak up the medium at the edge. The inner pool of the Dunn chamber (Hawksley, Sussex, UK) was filled with medium and CCL19 chemokine (400 ng/mL, R&D systems); the outer pool was filled with medium only. We have also tested inverted combination in order to avoid loading affects. In that case, both inner and outer pools were at the first step filled with medium only and the content of outer pool has been subsequently replaced by fresh chemokine-supplemented medium. The results of MEC1 cells migration analysis from both tested loading schemes were comparable, For experiments with inhibitors CK1 inhibitor D4476 (100 μM) or PF670462 (50 μM) was added to both compartments. The inverted cover-slip was placed on the chamber and the outer well was refilled with fresh medium exactly according to manufacturer’s instructions. For 1 hour experiments, no hot wax mixture was placed to seal the cover slip. The filled chamber was then placed into the prewarmed microscope chamber at 5% CO_2_.

Glass-bottom plate/4-compartment glass-bottom plate/Ibidi chambers were coated with human plasma fibronectin and MEC1 cells were stained and seeded as described above. In 4-compartment chambers, stimulation of MEC1 cells was performed at the beginning of each experiment and 4 positions were scanned at the same time in total for 30 min. In glass-bottom plates, non-stimulated cells were captured for 30 min as control. Afterwards, CCL19 chemokine was added and cells were captured for another 30 min. Subsequently, cells were treated with the CK1 inhibitor PF 670462 and scanned again for 30 min. Ibidi chambers allowed to capture control sample and CCL19-treated sample for 30 min two different positions simultaneously. Afterwards, chemokine was added again to both chambers and in one of the stimulated chambers the CK1 inhibitor was added. Both positions were scanned for 30 min.

Images were captured on a Zeiss LSM710 confocal microscope. Data were further analyzed using ImageJ (NIH) software. Cell-tracking analysis was performed using the Bitplane Imaris Software 7.4 by tracking the red signal. In order to avoid analysis of dead or permanently immobilized cells, cells for tracking were selected manually. For the analysis of migration properties of tracked cells, we picked 4 parameters: track length, displacement length, maximum speed and straightness. Track length displays and measures the exact path of a tracked cell. Displacement length shows the shortest path between the starting and the end point of a tracked cell. Furthermore, Bitplane Imaris Software provides the information about maximum speed for each cell and performs specific tracking, which depicts the straightness of the cell track (ranging from 0 to the maximum 1).

### Transwell assays

The chemotaxis assay was conducted in HTS Transwell-96 well plates (Corning Incorporated) with 5.0 μm pore size polycarbonate membranes following the manufacturer’s instructions.

Total number of 0.3 × 10^6^ MEC1 cells were seeded in the upper well of the transwell plate. Chemokine gradient was created by addition of CCL19 chemokine (R&D Systems, CCL19/MIP-3beta, 361-MI) in concentration of 100 ng/mL for D4476 dose response testing and 200 ng/mL in case of VANGL2-Venus or VANGL2 siRNA testing to the lower well of the plate. Sterile PBS/0.1% BSA solution in corresponding amount was used in control conditions. Cells were incubated for 6 hours and then number of migrated cells was analyzed by Accuri C6 Flow Cytometer (BD Biosciences). The assessment of cell viability was performed by TMRE staining (Tetramethylrhodamine ethyl ester perchlorate, Sigma-Aldrich) as described previously [10]. The migration index was calculated as the number of cells (treated or untreated) migrating in response to the chemokine divided by the number of cells migrating toward the control medium only. Graphs show either migration index or number of migrated cells in 20 μL of sample taken from the lower well of the transwell plate.

### Statistics

Statistical analysis was performed using GraphPad Prism5 (GraphPad Software Inc., La Jolla, CA, USA). Nonlinear regression was performed in Graph Pad Prism5 using curve fitting (inhibitory dose response) with variable slope parameters.

To assess differences in more than two variables, data were tested by one-way ANOVA and Kruskal-Wallis test (*, P < 0.05; **, P < 0.01; ***, P < 0.001). Directionality of cell migration was analyzed using Oriana (Kovach Computing Services, UK) and Rayleigh’s test.

## Additional files

Additional file 1:
**Movie of migrating MEC1 cells.** Cell tracker (red) stained MEC1 cells were observed by confocal microscopy on a fibroenectin-coated glass surface. Note the distinct polarization of the cells. Additional file 2:
**3D-model of migrating MEC1 cell transfected with VANGL2-EGFP and actin-RFP.** A VANGL2-EGFP and actin-RFP cotransfected MEC1 cell was imaged employing the Z-stack function of the Zeiss ZEN software. #D rendering was done with the Imaris 7.6 software. Size bar 10 μm. Additional file 3:
**The effect of cytochalasin D treatment on the distribution of VANGL2-EGFP and the morphology of MEC1 cells.** A living MEC1 cells with distinct polarized VANGL2-EGFP expression on a fibronectin-coated glass bottom dish was imaged by laser scanning microscopy. After 22 min the cells were treated with cytochalasin D, a potent inhibitor of actin polymerization. Note the rapid morphological changes in MEC1 cells from a polarized migratory phenotype to a rounded morphology. Simultaneously, the polarized VANGL2-EGFP distribution is maintained arguing that polarized distribution of VANGL2 is a cause for and not a consequence of asymmetric shape of a migrating cell.

## Abbreviations

CLL: chronic lymphocytic leukemia
CCL19: chemokine (C-C motif) ligand 19
CK1: Casein kinase 1
MEC1: Patient-derived B-chronic lymphocytic leukemia cell line spontaneously grown from peripheral blood lymphocytes
PCP: Planar cell polarity
VANGL2: Van Gogh-like 2
WNT: Wingless/*Int-1*
